# Fourteen-Day Bactericidal Activity, Safety, and Pharmacokinetics of Linezolid in Adults with Drug-Sensitive Pulmonary Tuberculosis

**DOI:** 10.1128/AAC.02012-19

**Published:** 2020-03-24

**Authors:** Andreas H. Diacon, Veronique R. De Jager, Rodney Dawson, Kim Narunsky, Naadira Vanker, Divan A. Burger, Daniel Everitt, Frances Pappas, Jerry Nedelman, Carl M. Mendel

**Affiliations:** a Division of Pulmonology, Department of Medicine, Faculty of Medicine and Health Sciences, Stellenbosch University, Cape Town, South Africa; b Task Applied Science, Bellville, South Africa; c Division of Pulmonology, Department of Medicine, Groote Schuur Hospital and University of Cape Town Lung Institute, Cape Town, South Africa; d Task Laboratory, Parow, South Africa; e Department of Statistics, University of Pretoria, Pretoria, South Africa; f TB Alliance, New York, New York, USA

**Keywords:** *Mycobacterium tuberculosis*, bactericidal activity, linezolid

## Abstract

Linezolid is increasingly used for the treatment of tuberculosis resistant to first-line agents, but the most effective dosing strategy is yet unknown. From November 2014 to November 2016, we randomized 114 drug-sensitive treatment-naive pulmonary tuberculosis patients from Cape Town, South Africa, to one of six 14-day treatment arms containing linezolid at 300 mg once daily (QD), 300 mg twice daily (BD), 600 mg QD, 600 mg BD, 1,200 mg QD, 1,200 mg three times per week (TIW), or a combination of isoniazid, rifampin, pyrazinamide, and ethambutol. Sixteen-hour sputum samples were collected overnight, and bactericidal activity was characterized by the daily percentage change in time to positivity (TTP) and the daily rate of change in log_10_(CFU). We also assessed the safety and pharmacokinetics of the study treatments. We found that bactericidal activity increased with increasing doses of linezolid. Based on the daily percentage change in TTP, activity was highest for 1,200 mg QD (4.5%; 95% Bayesian confidence interval [BCI], 3.3 to 5.6), followed by 600 mg BD (4.1%; BCI, 2.5 to 5.7), 600 mg QD (4.1%; BCI, 2.9 to 5.3), 300 mg BD (3.3%; BCI, 1.9 to 4.7), 300 mg QD (2.3%; BCI, 1.1 to 3.5), and 1,200 mg TIW (2.2%; BCI, 1.1 to 3.3). Similar results were seen with bactericidal activity characterized by the daily rate of change in CFU count. Antimycobacterial activity correlated positively with plasma drug exposure and percentage time over MIC. There were no unexpected adverse events. All linezolid doses showed bactericidal activity. For the same total daily dose, once-daily dosing proved to be at least as effective as a divided twice-daily dose. An intermittent dosing regimen, with 1,200 mg given three times weekly, showed the least activity. (This study has been registered at ClinicalTrials.gov under identifier NCT02279875.)

## Abstract (precis)

Linezolid is increasingly used for the treatment of tuberculosis resistant to first-line agents, but the most effective dosing strategy is yet unknown. From November 2014 to November 2016, we randomized 114 drug-sensitive treatment-naive pulmonary tuberculosis patients from Cape Town, South Africa, to one of six 14-day treatment arms containing linezolid at 300 mg once daily (QD), 300 mg twice daily (BD), 600 mg QD, 600 mg BD, 1,200 mg QD, 1,200 mg three times per week (TIW), or a combination of isoniazid, rifampin, pyrazinamide, and ethambutol.

## INTRODUCTION

Tuberculosis (TB) continues to be a major public health problem globally, with the number of persons infected with TB resistant to first-line drug therapy continuing to increase (1). Together with the novel nitroimidazole class of anti-TB agents, pretomanid and delamanid, and the novel diarylquinoline bedaquiline, linezolid is a good candidate for inclusion in a much-needed new regimen for treatment of drug-resistant TB. Previous studies have shown that linezolid may play a valuable role in improving the rate of culture conversion in patients with drug-resistant TB (2). In a prospective, randomized, controlled trial performed in South Korea, 87% of patients with extensively drug-resistant TB, unresponsive to TB chemotherapy within 6 months prior to study enrollment, achieved sputum culture conversion within 6 months after linezolid (600 mg/day) was added to their background regimen (2). Four (11%) patients acquired drug resistance to linezolid in this study, three of whom showed relatively low exposures of linezolid.

Despite the inclusion of linezolid in current drug-resistant TB regimens (3), there is limited information about the relationship between the dose of linezolid and its mycobactericidal activity. While linezolid is approved at a dose of 600 mg every 12 h for up to 28 days to treat selected bacterial infections (4), the toxicities of myelosuppression and peripheral neuropathy, in particular, have raised concerns about using this dose long term for the treatment of TB infections. Key toxicities of linezolid are thought to be related to inhibition of mitochondrial protein synthesis, and drug exposures above a threshold for this inhibition may have greater risk of causing toxicity. In reports of the use of linezolid beyond 2 months to treat TB, adverse events were primarily related to hematological, neurological, and gastrointestinal disorders. Based on a review of the literature, hematological disorders were generally moderate and reversible upon discontinuation of linezolid. Peripheral neuropathy often resolved or partially resolved with dosage reduction or discontinuation of linezolid, although cases of irreversible peripheral neuropathy have been reported (5–8). A systematic review and meta-analysis of prolonged use of linezolid found that myelosuppression occurred in a higher proportion of patients than neuropathy but that in most studies this could be managed by temporarily or permanently discontinuing linezolid therapy (9). The authors also reported that the incidence of myelosuppression was dose related, with lower doses being associated with lower incidence, while neuropathy was not highly associated with higher doses of linezolid.

Preclinical studies in a mouse model of infection have shown that drug exposure equivalent to a 1,200 mg total daily dose in humans is required to produce mycobacterostatic activity (10). Consequently, this study was undertaken to provide rigorous information about the bactericidal activity of various dosing schemes in humans with new TB infections over the first 14 days of treatment. The results will allow a better assessment of the potential risks and benefits as linezolid continues to be incorporated in TB treatment regimens.

## RESULTS

### Participants.

**FIG 1:**
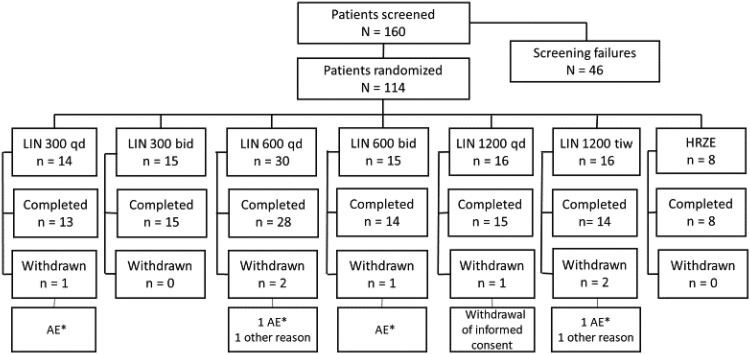
Participant disposition. One participant (receiving linezolid at 1,200 mg TIW) died from massive hemoptysis (not related to study treatment). Four participants (one each receiving 300 mg QD, 600 mg QD, 600 mg BD, and 1200 mg TIW) were withdrawn for elevations in liver enzymes (alanine aminotransferase [ALT] and/or aspartate aminotransferase [AST]) that reached grade 4 in two cases and grade 3 in another two cases. Two participants withdrew consent from further study participation (1,200 mg QD and 600 mg QD) for personal reasons not related to an adverse event. LIN, linezolid; QD, once daily; BID, twice daily; TIW, three times weekly; AE, adverse event. HRZE is a fixed-dose combination of isoniazid, rifampin, pyrazinamide, and ethambutol.

Of the 114 enrolled participants, 107 completed the study up to the final follow-up visit. Seven participants were withdrawn from the study (Fig. 1). Among all enrolled participants, the mean age was 33 years, 83% were male, and 96% were HIV negative (Table 1).

**TABLE 1:** Baseline characteristics of study participants^*a*^

Treatment group	No. of participants	No. of males (%)	No. of mixed ethnicity (%)^*b*^	Age (yrs) (mean [SD])	BMI (kg/M^2^) (mean [SD])	No. of HIV positive (%)	Baseline CFU (log_10_/ml sputum)		Baseline log_10_(TTP) (h)	
							Mean	SD	Mean	SD
LIN, 300 QD	14	12 (85.7)	11 (78.6)	29.7 (7.66)	18.94 (1.755)	0	5.931	1.460	2.033	0.106
LIN, 300 BD	15	14 (93.3)	10 (66.7)	30.7 (11.32)	19.38 (2.549)	0	5.469	1.218	2.064	0.134
LIN, 600 QD	30	22 (73.3)	14 (46.7)	33.1 (10.63)	18.99 (2.181)	1 (3.3)	6.272	0.876	2.027	0.096
LIN, 600 BD	15	13 (86.7)	7 (46.7)	32.3 (10.44)	18.45 (2.572)	0	5.424	1.632	2.061	0.155
LIN, 1,200 QD	16	13 (86.7)	8 (53.3)	34.0 (14.44)	20.00 (3.339)	2 (13.3)	5.769	1.391	2.068	0.184
LIN, 1,200 TIW	16	13 (81.3)	6 (37.5)	35.1 (12.81)	19.39 (2.882)	0	6.172	0.947	2.046	0.105
HRZE	8	7 (87.5)	3 (37.5)	35.9 (8.97)	19.70 (3.829)	1 (12.5)	6.046	0.803	2.070	0.085
All	114	94 (83.2)	59 (52.2)	32.9 (11.06)	19.21 (2.619)	4 (3.5)	5.869	1.189	2.052	0.123

a BMI, body mass index; HIV, human immunodeficiency virus; LIN, linezolid; TTP, time to positivity; HRZE, fixed-dose combination of isoniazid, rifampin, pyrazinamide, and ethambutol; SD, standard deviation.

b Mixed ethnicity refers to the multiracial ethnic group native to Southern Africa.

### Bactericidal activity.

**FIG 2:**
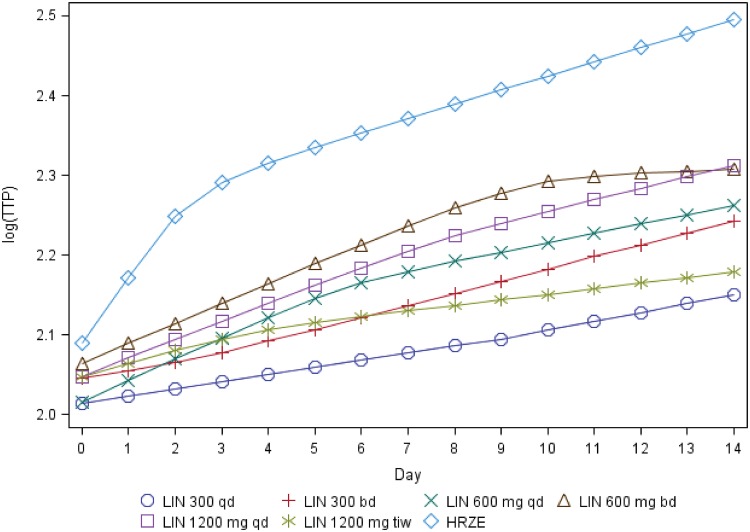
Posterior estimates of mean log_10_(TTP) over 14 treatment days. Abbreviations are as described for Fig. 1.

**FIG 3:**
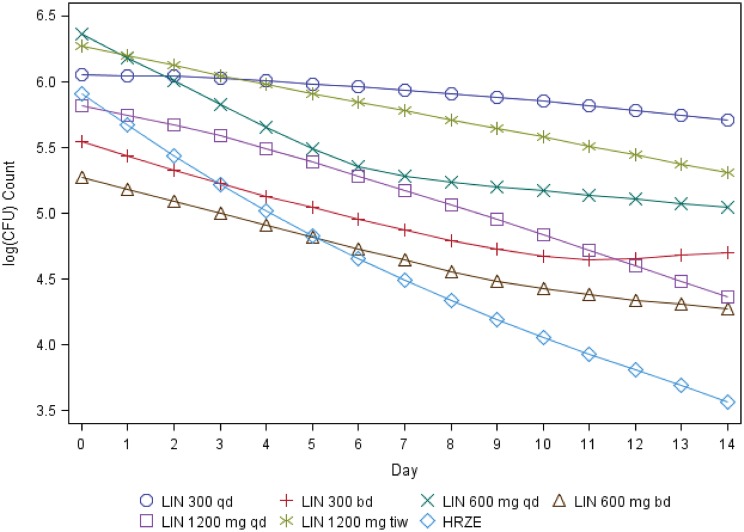
Posterior estimates of mean log_10_(CFU) count over 14 treatment days. Abbreviations are as described for Fig. 1.

The highest mean bactericidal activity over 14 days was seen with the highest once-daily (QD) dose of linezolid, 1,200 mg, while 600 mg QD and 300 mg QD had lower mean activities (Fig. 2 and 3). Confidence intervals for mean responses of the dose groups overlapped (Tables 2 and 3), but the Jonckheere-Terpstra (J-T) test was significant (*P* < 0.001), providing evidence for an ordered dose response. For those receiving the same total daily dose, twice-daily dosing did not show any advantage over once-daily dosing. The two linezolid 600 mg QD groups, analyzed separately and pooled, showed similar bactericidal activity for both daily percentage change in time to positivity (TTP) and CFU. The pooled data in the final analysis thus resulted in a group size twice that of the other linezolid groups. The control group receiving standard therapy (isoniazid, rifampin, pyrazinamide, and ethambutol [HRZE]) showed the expected change in viable mycobacterial load for both bactericidal activity endpoints, change over time in TTP, and CFU count. All infecting bacteria were identified as Mycobacterium tuberculosis, and all patients on HRZE were susceptible to all anti-TB agents.

**TABLE 2:** Bactericidal activity per treatment arm expressed as the daily percentage change in TTP from day 0 to day 14^*a*^

Treatment arm	No. of participants	Posterior estimate (95% BCI) for days:		
		0–14	0–2	7–14
LIN, 300 QD	14	2.269 (1.071; 3.535)	2.073 (–0.535; 4.840)	2.477 (–0.375; 5.270)
LIN, 300 BD	15	3.303 (1.949; 4.663)	2.268 (–3.809; 8.091)	3.557 (1.945; 5.083)
LIN, 600 mg QD	30	4.128 (2.943; 5.342)	6.392 (4.763; 8.173)	2.746 (1.032; 4.505)
LIN, 600 mg BD	15	4.071 (2.521; 5.666)	5.962 (3.759; 8.517)	2.337 (0.171; 4.593)
LIN, 1200 mg QD	15	4.458 (3.301; 5.630)	5.518 (2.729; 8.156)	3.585 (2.127; 5.156)
LIN, 1200 mg TIW	15	2.178 (1.101; 3.253)	3.832 (1.401; 6.598)	1.595 (0.232; 2.889)
HRZE	8	6.918 (4.825; 9.141)	20.189 (10.200; 30.240)	4.174 (1.755; 6.685)

a BCI, Bayesian credibility interval; LIN, linezolid; TTP, time to positivity; HRZE, fixed-dose combination of isoniazid, rifampin, pyrazinamide, and ethambutol.

**TABLE 3:** Bactericidal activity per treatment arm expressed as the daily rate of change in log_10_(CFU) count from day 0 to day 14^*a*^

Treatment arm	No. of participants	Posterior estimate (95% BCI) for days:		
		0–14	0–2	7–14
LIN, 300 QD	14	0.024 (–0.020; 0.071)	0.006 (–0.138; 0.116)	0.032 (–0.038; 0.103)
LIN, 300 BID	15	0.060 (0.008; 0.114)	0.110 (0.026; 0.230)	0.025 (–0.054; 0.100)
LIN, 600 mg QD	30	0.094 (0.060; 0.126)	0.177 (0.121; 0.238)	0.035 (–0.021; 0.090)
LIN, 600 mg BID	15	0.072 (0.014; 0.127)	0.093 (0.008; 0.178)	0.053 (–0.031; 0.133)
LIN, 1,200 mg QD	15	0.104 (0.052; 0.158)	0.071 (–0.071; 0.189)	0.116 (0.048; 0.188)
LIN, 1,200 mg TIW	15	0.069 (0.034; 0.105)	0.076 (–0.051; 0.191)	0.067 (0.023; 0.112)
HRZE	8	0.167 (0.088; 0.245)	0.238 (0.096; 0.457)	0.132 (0.029; 0.228)

a BCI, Bayesian credibility interval; LIN, linezolid; HRZE, fixed-dose combination of isoniazid, rifampin, pyrazinamide, and ethambutol.

### Safety and toxicity.

All adverse events seen were expected for linezolid from experience in other indications. The most commonly reported adverse events included rash and pruritus (8.8% each), diarrhea (7.1%), vomiting (5.3%), headache (4.4%), dizziness (3.5%), and increases in liver transaminases (4.4%) and amylase (5.3%). During the trial, 71 treatment-related adverse events were recorded, the majority of which were mild or moderate in severity. Adverse events were evenly distributed among the dose groups, with no obvious relationship between linezolid exposure/dose and number of events. Participants dosed only three times per week (linezolid at 1,200 mg TIW) experienced no less serious or frequent adverse events than participants receiving daily or twice-daily linezolid. As expected, critical events related to myelosuppression or neuropathy were not seen over the relatively short 2-week treatment period.

Of the 114 participants who started on linezolid, 4 (3.5%) were withdrawn due an adverse event (Fig. 1), 2 of whom developed a grade 3 elevation in transaminases (600 mg QD) and drug-induced liver injury (DILI) (600 mg BD), and another 2 developed grade 4 elevated transaminases (300 mg QD) and DILI (1,200 mg TIW). One participant in the 1,200 mg TIW group died early in treatment due to massive hemoptysis. Two participants withdrew consent from further study participation for reasons not related to an adverse event.

In terms of mitochondrial protein synthesis (MPS) inhibition, a clear dose-response relationship was observed. The twice-daily treatment regimens had higher mean time over MPS 50% inhibitory concentrations (IC_50_) than their once-daily counterparts with the same total daily dose, as follows: 99.9% for 600 mg BD, 77.3% for 300 mg BD, 88.9% for 1,200 mg QD, 54.8% for 600 mg QD, 24.8% for 300 mg QD, and 38.6% for 1,200 mg TIW.

### Pharmacokinetics and pharmacodynamics.

Over the 300 mg to 1,200 mg dose range, plasma maximum concentration of drug in serum (*C*_max_) and area under the concentration-time curve (AUC) increased more than proportionally to the dose (Table 4). The half-life also increased with the dose, consistent with the nonlinear pharmacokinetics of linezolid previously observed by some investigators (11–14) but not all (15, 16). In comparison to once-daily regimens with the same total daily dose, twice-daily administration provided comparable AUC, lower *C*_max_, and higher trough concentrations. There were no consistent gender effects. The MIC values of 91 participants treated with linezolid (86.7%) were 0.25, 0.5, and 1 μg/ml in 10, 61, and 20 participants, respectively, which is within the expected range of 0.125 to 1 μg/ml (17). Linezolid at 1,200 mg QD, 300 mg BD, and 600 mg BD reached 100% time above MIC in all participants, while 300 mg QD and 600 mg QD reached a mean of 58% and 89% time above MIC, respectively. Spearman correlation coefficients with TTP were positive and statistically significant (*P* < 0.05) for *C*_max_, average concentration of drug in serum (*C*_avg_), AUC from 0 to 24 h [AUC_(0–24)_], percentage time over MIC (*T*_MIC_), time over MPS IC_50_, and *C*_avg_/MIC and were moderately large in magnitude (>0.4) for *C*_avg_, *T*_MIC_, and time over MPS IC_50_. These findings suggest that linezolid has concentration-dependent bactericidal activity against M. tuberculosis.

**TABLE 4:** Pharmacokinetics of linezolid^*a*^^,^^*b*^

Parameter (unit)	LIN, 300 mg QD (*n* = 13)^*c*^	LIN, 600 mg QD (*n* = 28)^*d*^	LIN, 1,200 mg QD (*n* = 15)	LIN, 300 mg BD (*n* = 15)	LIN, 600 mg BD (*n* = 14)	LIN, 1,200 mg TIW (*n* = 13)
*C*_max_ (mcg/ml)	7.056 (21.1)	14.46 (23.9)	30.25 (20.5)	9.266 (29.6)	23.92 (20.9)	26.01 (25.4)
*T*_max_ (h)^*e*^	1.03 (0.50–4.00)	2.00 (0.50–4.03)	1.00 (0.50–2.02)	1.00 (0.50–2.05)	1.01 (0.50–2.00)	2.00 (0.92–4.00)
AUC_(0–tau)_ (mcg × h/ml)	40.67 (35.5)	106.8 (36.5)	287.7 (30.4)	54.90 (24.7)	167.9 (26.8)	244.7 (37.3)
AUC_(0–24)_ (mcg × h/ml)	40.67 (35.5)	106.8 (36.5)	287.7 (30.4)	109.7 (24.7)	335.6 (26.8)	228.4 (31.1)
AUC_(0–168)_ (mcg × h/ml)	ND	ND	ND	ND	ND	736.5 (37.7)
*C*_avg_ (mcg/ml)	1.694 (35.6)	4.450 (36.4)	12.00 (30.4)	4.602 (24.8)	14.08 (26.9)	4.386 (37.7)
*C*_(predose)_ (mcg/ml)	0.1653 (75.6)	0.5278 (147.0)	2.419 (83.8)	2.487 (38.7)	8.819 (42.1)	0.3134 (48.5)
*t*_1/2_ (h)	3.598 (31.3)	4.573 (38.6)	6.446 (28.8)	4.982 (23.3)	6.340 (28.5)	5.351 (45.4)

a LIN, linezolid; ND, not determined.

b Unless indicated otherwise, all values are given as the geometric mean and percent coefficient of variation (% CV).

c *n* = 11 for *t*_1/2_.

d Results of pooled data for participants recruited before and after amendment 02 of the protocol are presented for linezolid at 600 mg QD. *n* = 26 for *t*_1/2_.

e Median (minimum – maximum).

## DISCUSSION

In this 2-week monotherapy study with increasing doses of linezolid, the greatest antimycobacterial activity was found with the once-daily 1,200 mg dose, with lower activity seen when smaller daily doses were given. Twice-daily dosing appeared to have no clear advantage over once-daily dosing. Linezolid once-daily regimens also showed a lower mean percentage time over MPS IC_50_ and may thus be associated with relatively less toxicity over a prolonged treatment period.

The bactericidal activity of the highest tested dose, 1,200 mg daily, with a daily mean log_10_(CFU) decline of 0.104 (95% Bayesian confidence interval [BCI], 0.052 to 0.158), is in the range of that found previously for established anti-TB agents, such as rifampin at 10 mg/kg or pyrazinamide at 2 g (18), and more recently evaluated novel compounds, such as bedaquiline at 400 mg, pretomanid at 200 mg, meropenem-amoxicillin-clavulanic acid three times daily, and sutezolid at 600 mg BD (19–23). In an ongoing study of participants with highly drug-resistant TB treated with a combination of bedaquiline, pretomanid, and linezolid, an overall cure rate of approximately 90% has been observed, with this all-oral triple drug combination recently receiving U.S. FDA approval for use in this patient population (24).

Based on our study findings, higher doses of linezolid appeared more active, at least during the first 2 weeks of treatment, and the same total daily dose is at least as effective given once daily as when given in a divided twice-daily dose. As an alternative dosing strategy to exploit its early bactericidal potential while maintaining an acceptable toxicity profile, one may consider linezolid treatment initiation at the highest tested dose, 1,200 mg daily, for the first 2 to 4 weeks, followed by dose reduction to 600 mg or 300 mg once daily based on individual tolerability. However, further studies are needed to explore the long-term linezolid safety profile at such high doses, while the need for dose modifications should continue to be guided by individual risk/benefit assessments.

This study adds to the mounting evidence that the oxazolidinones continue to have their place in antituberculosis treatment regimens, provided that long-term toxicity can be managed.

## MATERIALS AND METHODS

### Participants.

The study was conducted at two sites in Cape Town, South Africa, the TASK Applied Science Tuberculosis Clinical Research Centre and the University of Cape Town Lung Institute. Local ethics and regulatory approvals were received prior to the conduct of the study. The study is registered at www.clinicaltrials.gov with the study identifier NCT02279875. From November 2014 to November 2016, we included 114 patients with rifampin-sensitive pulmonary TB showing at least 1+ positive for acid-fast bacilli on sputum microscopy (as per the WHO/International Union against Tuberculosis and Lung Disease scale). Participants were treatment-naive, aged between 18 and 75 years with body weight of 35 to 100 kg, had a chest X-ray compatible with pulmonary TB, and were able to produce at least 10 ml of sputum during a 16-h collection period. Patients with evidence of extrathoracic TB, poor general condition requiring immediate initiation of anti-TB therapy, diabetes mellitus, human immunodeficiency virus (HIV) infection with a CD4^+^ cell count of ≤250 cells/μl, or significant cardiac arrhythmias were excluded. After completion of the 14-day study treatment, all participants were started on standard-of-care anti-TB therapy and were followed up after 14 days to exclude late signs of toxicity and to ascertain that they were receiving standard TB treatment at their community clinics.

### Treatments.

Eligible participants were randomly assigned to one of five linezolid treatment groups with approximately 15 participants each—300 mg once daily (QD), 300 mg twice daily (BD), 600 mg QD, 600 mg BD, 1,200 mg QD—or a smaller control group receiving standard combination-drug therapy (weight-banded isoniazid, rifampin, pyrazinamide, and ethambutol [HRZE] combination tablets according to South African National TB Program guidelines). After completion of the once-daily and twice-daily dosing groups, two additional groups of approximately 15 participants each were included, receiving linezolid at 1,200 mg three times per week (TIW) or 600 mg QD (for temporal comparison with the previous group). Therapy was administered for 14 consecutive days by study staff 1 h before or 2 h after meals with 250 ml water at approximately the same time daily throughout the study period.

### Safety and toxicity.

Participants were hospitalized for the duration of the study treatment and assessed daily by the study physicians for adverse events that were graded according to the Division of Microbiology and Infectious Diseases adult toxicity table. Regular monitoring for specific laboratory toxicities was based on target organs defined in preclinical toxicity studies and included, among others, elevations in transaminases (alanine and aspartate aminotransferase), amylase, lipase, features of myelosuppression, and lactic acidosis. Adverse events were elicited by means of open-ended questions. Participants who experienced signs or symptoms of peripheral neuropathy, optic neuropathy, or seizures were to permanently discontinue study treatment and be managed according to standard medical practice. Impairment of human mitochondrial protein synthesis (MPS) is suspected to be the underlying mechanism associated with these common toxicities of long-term linezolid use. For each participant, we calculated the percentage of time the linezolid concentration exceeded MPS IC_50_, the concentration of linezolid required to inhibit 50% of MPS. The value of MPS IC_50_ we used was 2.7 μg/ml, the median of 25 independent assessments derived from an *in vitro* study (internal data).

### Microbiology.

Before participants were included in the study, susceptibility to rifampin was ascertained with the Genotype MTBDRplus line probe assay (Hain, Nehren, Germany). For endpoint assessments, sputum was collected for 16 consecutive hours overnight for 2 days prior to study therapy initiation and daily from day 1 to 14 during therapy. Sputum samples were kept at 2 to 8°C before transport to the central laboratory at the Department of Medical Biochemistry, Faculty of Health Sciences, Stellenbosch University, Cape Town. For time to positivity (TTP) measurement, sputum was homogenized by magnetic stirring, decontaminated with NaOH-NALC (AlphaTec NAC-PAC Red; AlphaTec, Vancouver, WA, USA), and incubated in duplicate in a standardized liquid culture system (Bactec MGIT 960; BD, Franklin Lakes, NJ, USA). TTP was recorded in hours. Additionally, homogenized nondecontaminated sputum was inoculated in 10-fold dilutions in quadruplicate onto 7H11S agar plates (BD) made selective with the addition of Selecatab (MAST, Merseyside, UK) and incubated for 3 to 4 weeks for CFU counting. Phenotypical susceptibility to first-line anti-TB agents was ascertained with the mycobacterial growth indicator tube (MGIT) system (BD). MICs of participants’ isolates to linezolid were assessed with the agar proportion method.

### Pharmacokinetics and pharmacodynamics.

Blood draws for pharmacokinetic measurements occurred on day 14 of study therapy for all participants in the linezolid-containing treatment groups. Sampling occurred predose and at 0.5, 1, 2, 4, 8, 12, and 24 h postdose. At each time point, approximately 9 ml of blood was collected in a lithium-heparin blood collection tube, placed on ice, and centrifuged within 45 min of collection in a refrigerated centrifuge at 1,500 × *g* for 10 min. Plasma was then transferred into two polypropylene tubes and stored at –20°C until shipment and analyzed using a validated method. Pharmacodynamics were assessed by determining the percentage time over MIC (*T*_MIC_).

### Statistical analyses.

This was an observational study with no prespecified hypothesis testing. Bactericidal activity was characterized by the daily percentage change in TTP and the daily rate of change in log_10_(CFU) count over 14 treatment days using Bayesian nonlinear mixed effects regression modeling (25, 26). This model was designed specifically for this type of data and can handle missing data due to contamination, early participant withdrawal, or culture conversion. The model accounts for correlations between the random intercepts and slopes over time (e.g., bacterial load at baseline is typically associated with the rate of change in CFU count or TTP over time [27]). Dose response was assessed using the Jonckheere-Terpstra (J-T) test (28). For safety and tolerability endpoints, we determined the incidence and severity of adverse events. Pharmacokinetic parameters included maximum linezolid plasma concentration (*C*_max_), time of *C*_max_ (*T*_max_), area under the plasma concentration-time curve over the dosing intervals [AUC_(0–tau)_], from zero to 24 h [AUC_(0–24)_], and until 168 h [AUC_(0–168)_] for the three-times-weekly dosing group, as well as the average plasma concentration over the total dosing interval (*C*_avg_). Spearman correlation coefficients were evaluated between 14-day bactericidal activity and *C*_max_, *C*_avg_, AUC_(0–24)_, TMIC, time over MPS IC_50_, *C*_max_/MIC, *C*_avg_/MIC, and AUC_(0–24)_/MIC. Pharmacokinetic (PK) indices were calculated using Phoenix WinNonlin (Certara, Princeton, NJ, USA).
